# Two distinct superconducting phases in LiFeAs

**DOI:** 10.1038/srep27926

**Published:** 2016-06-14

**Authors:** P. K. Nag, R. Schlegel, D. Baumann, H.-J. Grafe, R. Beck, S. Wurmehl, B. Büchner, C. Hess

**Affiliations:** 1 IFW Dresden, Institute for Solid State Research, 01171 Dresden, Germany; 2 Institute for Solid State Physics, TU Dresden, 01069 Dresden, Germany; 3 Center for Transport and Devices, TU Dresden, 01069 Dresden, Germany

## Abstract

A non-trivial temperature evolution of superconductivity including a temperature-induced phase transition between two superconducting phases or even a time-reversal symmetry breaking order parameter is in principle expected in multiband superconductors such as iron-pnictides. Here we present scanning tunnelling spectroscopy data of LiFeAs which reveal two distinct superconducting phases: at 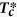 = 18 K a partial superconducting gap opens, evidenced by subtle, yet clear features in the tunnelling spectra, i.e. particle-hole symmetric coherence peak and dip-hump structures. At *T*_*c*_ = 16 K, these features substantiate dramatically and become characteristic of full superconductivity. Remarkably, the distance between the dip-hump structures and the coherence peaks remains practically constant in the whole temperature regime*T* ≤ 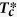. This rules out the connection of the dip-hump structures to an antiferromagnetic spin resonance.

The physics of LiFeAs seems to differ in many aspects from that of canonical iron-based superconductors, and accordingly attracts considerable attention. Unlike the latter, where superconductivity emerges from a Fermi surface-nested antiferromagnetic spin density wave (SDW) state upon doping^1^,^2^,^3^,^4^, LiFeAs superconducts without any doping^5^. It is thus ideally suited to experimentally investigate superconductivity in pristine matter, because doping-induced disorder can be excluded. The fermiology of LiFeAs has been much under debate: after early de Haas-van Alphen (dHvA) and scanning tunnelling spectroscopy (STS) experiments from which a nested Fermi surface had been deduced^6^,^7^, there exists now a comprehensive data set from transport^8^, dHvA^9^, STS^10^,^11^, and inelastic neutron scattering (INS)^12^,^13^ experiments which fully support^11^,^14^ reports of a Fermi surface without any nesting from angular resolved photoemission spectroscopy (ARPES)^15^,^16^,^17^,^18^. These very precise ARPES data, which concern both the band and the gap structure, have very recently rendered the physical properties of LiFeAs a paradigmatic testbed for theoretical models on multiband superconductors^19^,^20^,^21^. In the framework of such analysis it has been argued that the superconducting order parameter may enter a time-reversal symmetry breaking state and/or exhibit a peculiar temperature dependence^21^. Interestingly, an unconventional temperature evolution seems partially supported by previous experimental works which reported the critical temperature *T*_*c*_ for stoichiometric LiFeAs with values that scatter between about 15 K and 18 K^8^,^22^,^23^,^24^,^25^,^26^,^27^,^28^,^29^,^30^. Furthermore, the occurrence of multiple critical temperatures has previously been reported from successive nuclear magnetic resonance (NMR) Knight shift and AC-susceptibility measurements of one LiFeAs single crystal^31^. However, in such experiments, which typically probe the bulk or the global surface of a superconductor, it is very difficult to rule out sample inhomogeneity which in principle could cause the mentioned probes to respond to different parts of the sample, each with a potentially different critical temperature.

In order to clarify the temperature dependence of superconductivity in LiFeAs, we performed temperature dependent STS on a spatially fixed 2 nm × 2 nm clean surface area of a LiFeAs single crystal. Since the lateral size of this area is of the order of the coherence length of this material^32^,^33^, the aforementioned complications due to thinkable spatial inhomogeneity of stoichiometry or superconductivity can be excluded. In addition, we took great care to guarantee a very high global stoichiometric homogeneity of the chosen crystal by confirming an unprecedented sharpness of the ^75^As line in nuclear quadrupole magnetic resonance (NQR), which gives a very good account of the global distribution of the electrical field gradient at the As sites and thus the chemical homogeneity. The most striking observation in our experiments is an apparent phase transition within the superconducting state with dramatic impact on the spectral signatures of superconductivity in the differential conductance *dI*/*dV*. More specifically, we observe the onset of faint superconductivity at 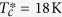 as evidenced by a particle-hole symmetric depletion and coherence peaks seen in the *dI*/*dV* at zero bias voltage and at about ±3–4 mV, respectively. In addition, characteristic dip-hump modifications in the *dI*/*dV* appear on both polarities at a distance of about 5–6 meV from the coherence peaks. These features affect only a tiny but well resolvable portion of the total *dI*/*dV*. Upon lowering the temperature (*T*), however, these features become dramatically enhanced at *T*_*c*_ = 16 K and rapidly develop into those of a fully gapped superconducting state upon further cooling. At the transition at 16 K, the coherence peaks shift to about ±6 mV which is already about 90% of the low-temperature gap value. Remarkably, the distance between the dip-hump structure and the coherence peak on either polarity stays largely unaffected from this transition. This unusual non-BCS-like temperature-evolution of superconductivity, in connection with recent ARPES, INS, and theory results, sets new constraints in understanding the peculiar superconducting state of LiFeAs.

## Results

**Figure 1:**
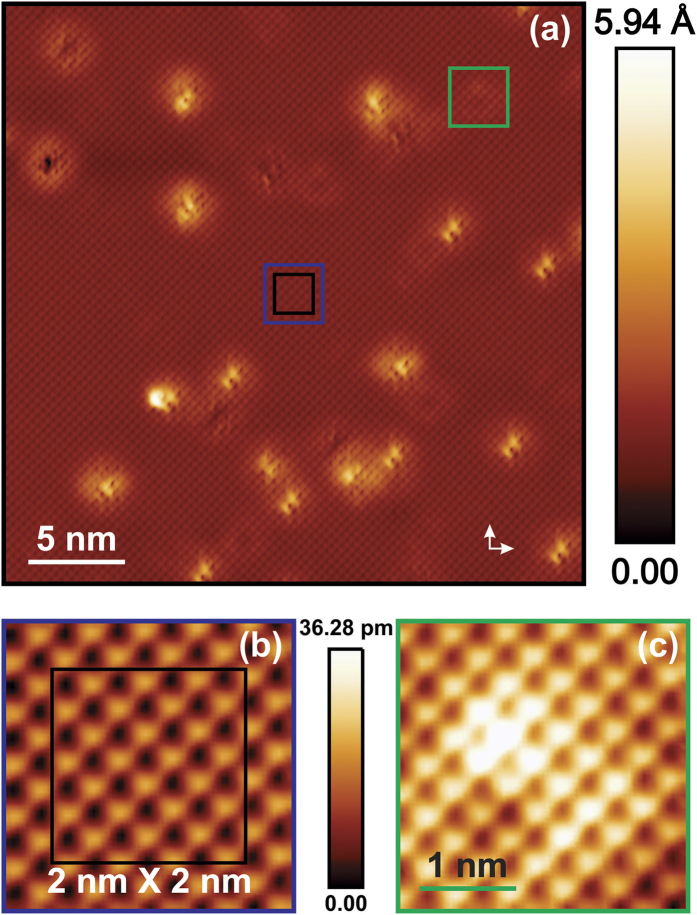
(**a**) 30 nm × 30 nm area of atomically resolved constant current mode topography image of LiFeAs (*I*_*T*_ = 300 pA, *V*_bias_ = +35 mV) measured at *T* = 4.8 K. White arrows indicate the in-plane shortest Fe-Fe directions. The atomic corrugation on the surface corresponds to the Li-Li (As-As) lattice spacing of 3.77 Å. 22 bright impurities from the first layer appear within the scan area. Faint signatures of impurities presumably of the second layer of the material are also visible (green square). Temperature dependent spectroscopy has been measured within the black square of 2 nm × 2 nm area. (**b**) Zoom-in into the blue square in (**a**) to show atomic contrast in absence of impurities. (**c**) Zoom-in into the green square in (**a**) to show the influence of an impurity in the second layer.

Figure 1a shows a representative topography scan with a field of view of 30 nm × 30 nm, measured at 4.8 K. The atomically resolved corrugation reveals the position of about 6300 surface Li-atoms^10^ with a lattice spacing of roughly 3.77 Å, matching the reported lattice constant of the material^5^. Isolated locations with bright contrast represent impurity states of the first layer of the material^34^. Beside these, faint structures appear in the image, presumably, arising from second layer impurity states as highlighted in Fig. 1c. In order to spectroscopically investigate the pristine electronic structure of the material we selected a 2 nm × 2 nm area (indicated by a black square area in Fig. 1a,b), far away from first and second layer impurity states, and performed STS as a function of temperature in this area.

**Figure 2:**
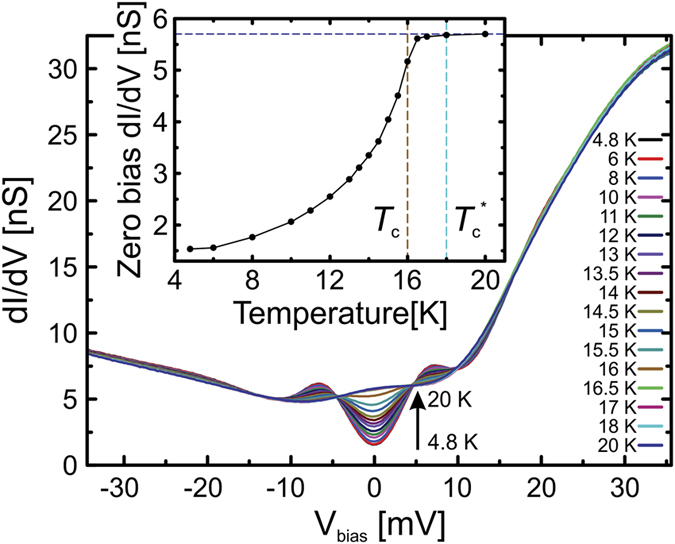
Temperature dependent tunnelling spectra measured within the black square of Fig. 1a,b between 4.8 K and 20 K. The up-arrow indicates the order of the curves at *V*_bias_ = 0 with increasing temperature. Inset: Zero bias differential conductance as a function of temperature. The horizontal dashed line is a guide to the eye. Vertical dashed lines indicate *T*_*c*_ and 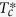, see text.

**Figure 3:**
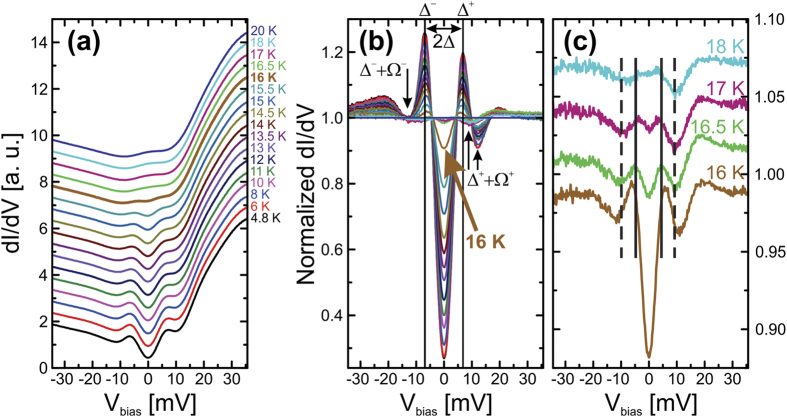
(**a**) Waterfall representation of the differential conductance *dI*/*dV* for various temperatures. The spectrum at 16 K is highlighted in bold. (**b**) Differential conductance *dI*/*dV* at various temperatures normalised to that at 20 K. Black up-arrows indicate the shift of the position of the positive energy dip at Δ^+^ + Ω^+^ towards lower energy upon raising the temperature through *T*_*c*_ = 16 K. The down-arrow indicates the coarse position of the negative energy dip at −Δ^−^ − Ω^−^. (**c**) Waterfall representation of normalised spectra in (**b**) at 16 K to 18 K. Superconducting coherence peaks and dip positions at 17 K are indicated by solid and dashed vertical lines, respectively.

Our results for the differential conductance *dI*/*dV* averaged within this area as a function of applied bias voltage *V*_bias_ for the different temperature levels are shown in Fig. 2. Two aspects can be recognised in the shown data: i) in the normal state (20 K) the *dI*/*dV* exhibits a strong asymmetry between occupied and unoccupied states with a hump-like enhancement around zero bias, in agreement with previous findings (Note that within literature and with respect to our data the coarse position of the hump varies by a few mV, presumably due to slightly different tunnelling matrix elements)^29^,^30^,^35^. ii) At base temperature of our system (4.8 K), pronounced signatures of the superconducting state are superimposed on the normal state *dI*/*dV* background, where the depletion of the *dI*/*dV* at zero bias and the appearance of coherence peaks at finite bias voltages are the most prominent indicators of the known largest superconducting gap Δ ≈ 6 meV in agreement with literature^17^,^18^,^29^,^30^,^35^. We mention that in spectroscopic studies at lower temperature (*T* < 6 K)^29^,^30^,^35^ further coherence peaks have been observed in accordance with smaller additional superconducting gaps observed by ARPES^17^,^18^. These become increasingly invisible in tunnelling spectra upon increasing the temperature due to thermal broadening, and therefore are not present in our low-temperature spectroscopic data at 6 K, as is consistent with the other tunneling data^29^,^30^,^35^. Upon increasing temperature, the data reveal a systematic closing of the gap with an apparent *T*_*c*_ of about 16 K (see also Fig. 3a). For illustrating the gap closing we plot the *dI*/*dV* at the energy of strongest depletion at zero bias in the inset of Fig. 2. The onset of a strong decrease of *dI*/*dV* below 16 K is clearly visible. However, a close inspection of the data (see inset of Fig. 2) reveals that the closing of the gap is incomplete even at temperatures *T* > 16 K.

In order to obtain further insight into the incomplete gap closing, we normalise all tunnelling spectra with respect to the normal state spectrum at 20 K, see Fig. 3b. In this representation, all features of the superconducting state become more pronounced. At small energies within the gap (as marked by the position of the coherence peaks) the spectra become almost particle-hole symmetric. Remarkably, this symmetry does not persist at larger energies. In particular, at positive *V*_bias_ a pronounced dip and a hump are present in the spectra at all temperatures (see Fig. 3b,c). However, at negative *V*_bias_, the dip corresponds to *dI*/*dV* values located barely below that of the normal state, whereas the hump structure is even more pronounced. Such dip-hump features resemble well-known tunnelling signatures of strong-coupling superconductivity^36^, and are frequently observed in different Fe-based superconductors, where these are often interpreted as fingerprints of spin fluctuations^29^,^37^,^38^,^39^. Surprisingly, the canonical spectral signatures of superconductivity, i.e. the coherence peaks, and the dip-hump structures are also clearly present in the normalised tunnelling data at temperatures *above* the afore-inferred *T*_*c*_ of 16 K. This becomes unambiguously clear in Fig. 3c which focuses on the temperature range 16 K to 18 K. Thus, in addition to the onset of pronounced superconductivity at *T*_*c*_ = 16 K, our LiFeAs sample is in a superconducting state that is characterised by faint corresponding spectral features already below 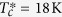.

**Figure 4:**
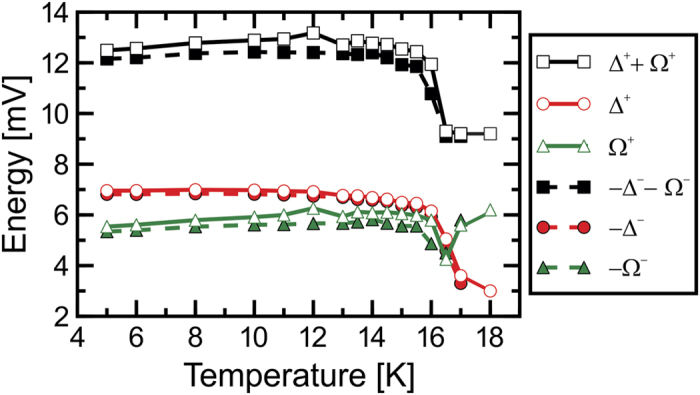
Temperature evolution of superconducting coherence peaks (Δ^+^, −Δ^−^), the dip positions (Δ^+^ + Ω^+^, −Δ^−^ − Ω^−^) and the resulting Ω^+^, −Ω^−^.

After having established this main experimental observation, we turn now to thoroughly analysing the observed spectral features as a function of temperature. In Fig. 3b we assign the distance between the coherence peaks at positive (negative) energy Δ^+^ (Δ^−^) to the double gap value 2Δ, and at each polarity the distance between the coherence peak and the dip position to the energy of a tentative bosonic mode Ω^+^ (Ω^−^) at positive (negative) energy. At base temperature (4.8 K) we find Δ ≈ 6.9 meV, and a practically particle-hole symmetric Ω^+^ ≈ |Ω^−^| = 5.4 ± 0.1 meV, consistent with previous findings^29^,^30^. The temperature evolution of the coherence peaks (Δ^+^, −Δ^−^), the dip positions (Δ^+^ + Ω^+^, −Δ^−^ − Ω^−^), and the mode energies (Ω^+^, Ω^−^) are summarised in Fig. 4. The peak positions which provide an estimate for the temperature dependence of the superconducting gap Δ(*T*), interestingly, remain almost constant up to almost *T*_*c*_. To be specific, Δ(*T*_*c*_ = 16 K)/Δ(4.8 K) ≈ 0.87. Upon increasing the temperature further, Δ(*T*) drops abruptly to about 50% of its low-temperature value at 17 K and becomes barely resolvable at 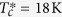. This unusual behaviour is in clear contrast to any BCS-like weak coupling scenario^40^ as has been previously suggested by Chi *et al*.^29^. Concerning the positions of the dips, a very similar temperature dependence is found in our data. These positions remain almost constant at a value of about ±12 mV up to *T*_*c*_ = 16 K, and jump-like decrease to about ±9 mV at higher temperature up to 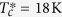. Remarkably, the energy of the tentative bosonic mode (Ω^+^, Ω^−^) exhibits only a small dip at around *T*_*c*_ but stays practically uninfluenced at lower and higher temperature.

## Discussion

The observation of two transition temperatures *T*_*c*_ and 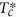 at an atomically fixed well-defined microscopic area of the order *ξ*^2^ reveals these as an intrinsic property of the material. Thereby it corroborates earlier findings where the measured critical temperature on the very same sample depended on the probing method and provides a reconciliation of the spread of reported *T*_*c*_ values^8^,^22^,^23^,^24^,^25^,^26^,^27^,^28^,^29^,^30^,^31^.

**Figure 5:**
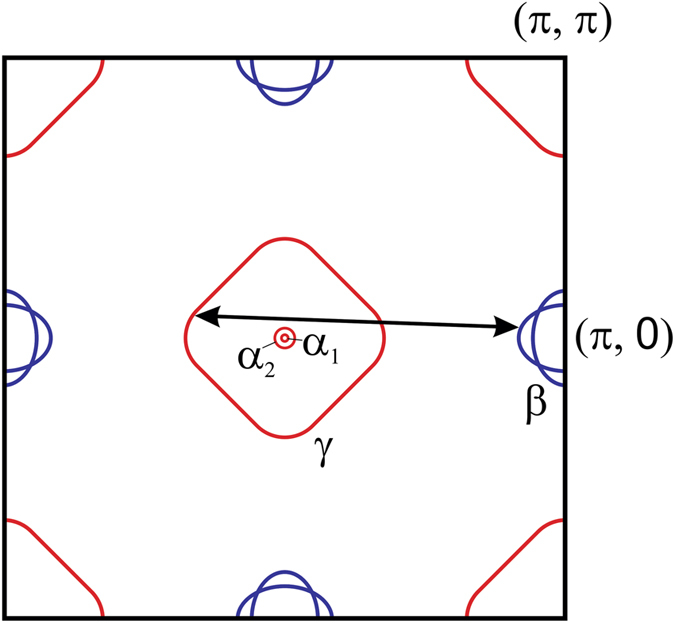
Schematic diagram of the first Brillouin zone (one-Fe unit cell) in LiFeAs based on ARPES data^19^. The indicated *γ*- and *β*- pockets possess only a weak *k*_*z*_-dispersion while the *α*_1_- and *α*_2_-pockets are located only close to *k*_*z*_ = *π*. The back arrow indicates the incommensurate spin fluctuation between the *γ*- and the *β*-bands observed by Qureshi *et al*.^12^,^13^,^14^.

A multiband electronic structure can in principle be pictured as a possible source for a complicated superconducting state with multiple order parameters^41^,^42^. In LiFeAs, according to the ARPES-derived electronic band structure^37^, the Fermi surface consists of quasi two-dimensional hole-like (labelled *γ*) and electron-like pockets (labelled *β*) centred around the Γ- and *M*-points, respectively (cf. Fig. 5). Two further hole-like Fermi surface pockets (labelled *α*_1_ and *α*_2_) are centred around the *Z*-point. The latter are tiny, yet have been reported to possess the largest superconducting gap Δ_*α*_ ≈ 6 meV as compared to Δ_*γ*,*β*_ = 3.5 to 4 meV at the *γ*- and *β*-pockets^17^,^18^. Recent theoretical work which focuses on analysing possible pairing scenarios on basis of this band structure suggests a complicated multigap order parameter which allows for peculiar temperature dependency^21^. In particular, it has been proposed that the *α*-Fermi surface pockets at *k*_*z*_ ≈ *π* may cause Cooper pairing prior to that of Fermi surface pockets at *k*_*z*_ ≈ 0, where the *α*-bands remain below the Fermi level. As a consequence, at temperatures just below the onset of superconductivity, the superconducting state may be very different from that at lower temperature, where all Fermi surface pockets contribute to the superconductivity. Considering such a scenario, the observed 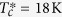 might be interpreted as the onset of superconductivity at *k*_*z*_ ≈ *π*, whereas the critical temperature *T*_*c*_ = 16 K can be related to the onset of full superconductivity emerging from the complete Fermi surface at all *k*_*z*_.

One might argue that in an alternative scenario the observed Δ at *T* > 16 K originates from the onset of superconductivity at the *γ*- and *β*-pockets, as Δ seemingly agrees with the measured low-temperature gaps Δ_*γ*,*β*_^17^,^18^,^29^,^30^,^35^, whereas the observed Δ at *T* ≤ 16 K results from the gap at the *α*-pockets, Δ_*α*_, after the onset of full superconductivity. While we cannot fully exclude this possibility, we consider this scenario to be less likely. The signatures of Δ_*γ*,*β*_ are well known to possess a strongly subordinate spectral weight in the tunneling spectra as compared to those of Δ_*α*_ which renders them practically invisible in spectroscopic data at 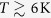
29,30. If the coherence peaks at *T* > 16 K stemmed from Δ_*γ*,*β*_ one would expect Δ_*α*_ to be almost closed at *T* = 16 K, giving rise only to a smaller relative spectral weight in the data approaching that of Δ_*γ*,*β*_ Thus, at this temperature, a double-gap feature or, considering the thermally broadened energy resolution at 16 K of about 3.5*k*_*B*_*T* ≈ 5 meV, at least significantly broadened peaks should be present in the normalized *dI*/*dV* data at 16 K. This is clearly not the case in the data.

The second unusual observation in our data is the practically temperature independent energy of the tentative bosonic mode Ω. In previous works, the dip indeed has been assigned to a strongly coupled bosonic mode connected to an antiferromagnetic spin resonance^29^,^35^. However, this interpretation can be clearly ruled out for the following reasons: Firstly, in case of a connection of the dip to an antiferromagnetic resonance, one expects that the mode energy Ω tracks the order parameter as a function of temperature. This is contradictory to our data. Secondly, an antiferromagnetic resonance at (*π*, 0) is, in fact, absent in LiFeAs^12^,^13^. Instead, INS reports only weak intensity response at incommensurate positions away from (*π*, 0), which has been assigned to ordinary interband excitations connecting poorly nested states in the *γ*- and the *β*-bands (see Fig. 5)^14^. This normal state feature of the dynamic spin susceptibility is too weak and too broad in energy to cause the very pronounced and sharp dip feature observed in the tunnelling spectra of the superconducting state.

Thus, our data reveal two important results: i) the origin of the dip-hump structure in the tunnelling spectra of LiFeAs is not related to an antiferromagnetic resonance. More thorough investigations in this regard are necessary for LiFeAs, and previous according interpretations for other iron-based superconductors^29^,^37^,^38^,^39^ should be carefully reconsidered. ii) The data reveal two distinct superconducting phases in LiFeAs, which underpins the unconventional nature of superconductivity in this compound. This calls for further investigations of its superconducting order parameter to verify other predicted unconventional properties such as breaking of time-reversal symmetry^21^,^43^.

## Methods

### Sample growth and characterisation

Single crystals of stoichiometric LiFeAs have been grown using the self-flux method as described by Morozov *et al*.^44^. In order to confirm stoichiometry and homogeneity of the sample prior to mounting it into our scanning tunnelling microscope (STM), we determined (see Supplementary Information) the ^75^As NQR frequency and line width of the sample as 21.54 MHz and 24 kHz, respectively. While the frequency is consistent with previous findings, the measured line width is by a factor of 1.5–3 times sharper than that of previously investigated samples^12^,^31^,^44^,^45^ highlighting the extraordinary homogeneity of our crystal. Since LiFeAs is highly air sensitive, these steps, and the actual mounting of the sample into our STM have been performed in Ar atmosphere.

### STM measurements

After mounting the sample into our home-built STM^46^, the sample space was evacuated, and cooled to about 5 K. Subsequently, the sample was cleaved in cryogenic vacuum just before approaching an electrochemically etched W-tip for performing the tunnelling measurements. Topography measurements have been executed in constant-current mode, while for measuring the *dI*/*dV* we used a lock-in amplifier with a modulation of 0.4 mV rms at 1.1111 kHz. In the used sign convention of data, negative *V*_bias_ probe the occupied states of the sample. In all shown spectroscopic data figures, an unavoidable slight offset of −0.6 mV has been corrected as to take account of the particle-hole symmetry of superconducting coherence peaks in the tunneling data. During the *dI*/*dV*-measurements at different temperatures we ensured that the tunnelling tip always probed the very same atoms within a 2 nm × 2 nm area to rule out any possible influence of sample inhomogeneity. Within this area, 800 grid spectra (including forward and backward sweep) have been recorded and averaged in the range ±35 mV with 0.35 mV energy point resolution. Thereby, the system was stabilised at −35 mV before and after every single forward and backward sweep. At each temperature level, we let the system sufficiently thermalise in order to have thermal drift less than an atom after every set of grid spectra (about 2 hours). Furthermore, we confirmed an unchanged tip state before and after each grid spectroscopy from comparing corresponding topography scans (see Supplementary Information). All images have been processed using the WSxM software^47^.

The coherence peaks and dip positions have been determined with a phenomenological parabolic fit to the corresponding extrema. The given errors are phenomenological estimates. Note, that Chi *et al*.^29^ use Dynes’ formula to extract the temperature dependence of two superconducting gaps in the system. We point out that such a procedure is not meaningful if the focus lies on extracting the subtle spectral features which occur near 16–18 K. Therefore, we restrict ourselves to analysing the direct temperature dependence of the observed salient spectral features only.

## Additional Information

**How to cite this article**: Nag, P. K. *et al*. Two distinct superconducting phases in LiFeAs. *Sci. Rep.* **6**, 27926; doi: 10.1038/srep27926 (2016).

## Supplementary Material

Supplementary Information
