# Supplementary material for: Two distinct superconducting phases in LiFeAs
Source: Sci Rep. 2016 Jun 14;6:27926. doi: 10.1038/srep27926 (PMC4906386; doi:10.1038/srep27926)
Supplement: Supplementary Information [file srep27926-s1.pdf]

Two distinct superconducting phases in LiFeAs: Supplementary Information

P. K. Nag,[1] R. Schlegel,[1] D. Baumann,[1] H.-J. Grafe,[1]
R. Beck,[1] S. Wurmehl,[1,2] B. Büchner,[1,2,3] and C. Hess[1,3]

[1]*IFW Dresden, Institute for Solid State Research, 01171 Dresden, Germany*

[2]*Institute for Solid State Physics, TU Dresden, 01069 Dresden*

[3]*Center for Transport and Devices, TU Dresden, 01069 Dresden, Germany*

(Dated: December 15, 2015)

Nuclear Quadrupole Resonance

Nuclear quadrupole resonance (NQR) measurements have been used to characterize the sample quality, i.e. the homogeneity of the LiFeAs single crystal. The ^{75}As NQR measurement was carried out at room temperature in Ar atmosphere inside a glove box prior to mounting the sample to the STM head. The results are shown in Fig. S1. The measured spectra have been fitted using a Gaussian function to extract the NQR frequency and the line width of the signal, which amount to 21.54 MHz and 0.024 MHz, respectively.

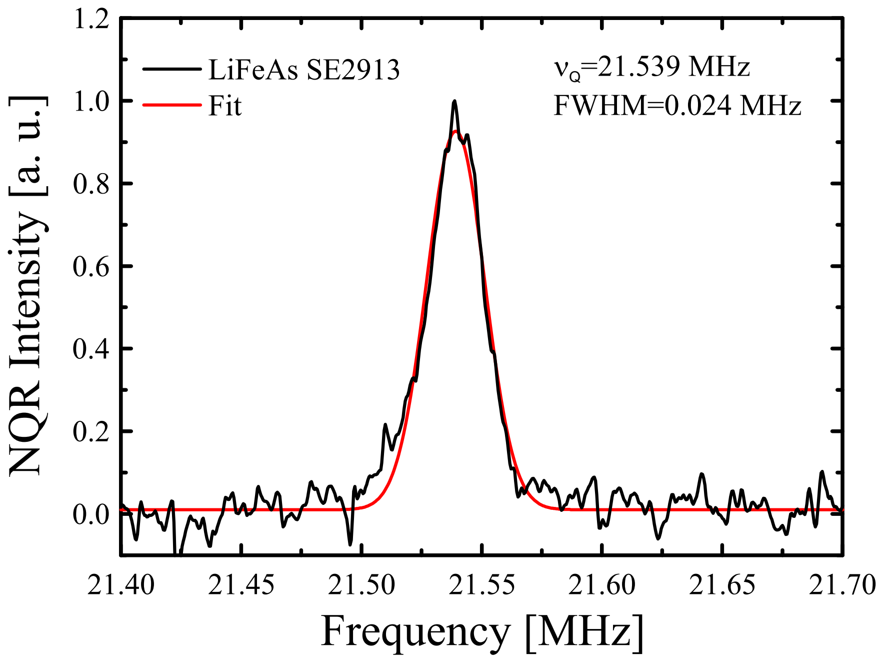

FIG. S1. Room temperature ^{75}As NQR measured on the LiFeAs single crystal inside Ar atmosphere. The red line represents a Gaussian fit to the data.

Temperature Dependent Tunneling Spectroscopy with Stable Tip State

The temperature dependent spectra have been measured on a defect free area as shown in the topography image of Fig. S2. The black box at the center of the images in Fig. S2a and b ensure a clean area far away from the influence of the defects (same as that in Fig. 1a of the paper).

Topography images for all different temperatures are shown in Fig. S3 demonstrating an unchanged tip state during the complete temperature dependent spectroscopy measurements.

An unchanged tip state is further demonstrated by the comparison of overview topography images at 4.8 K and 20 K shown in Fig. S4.

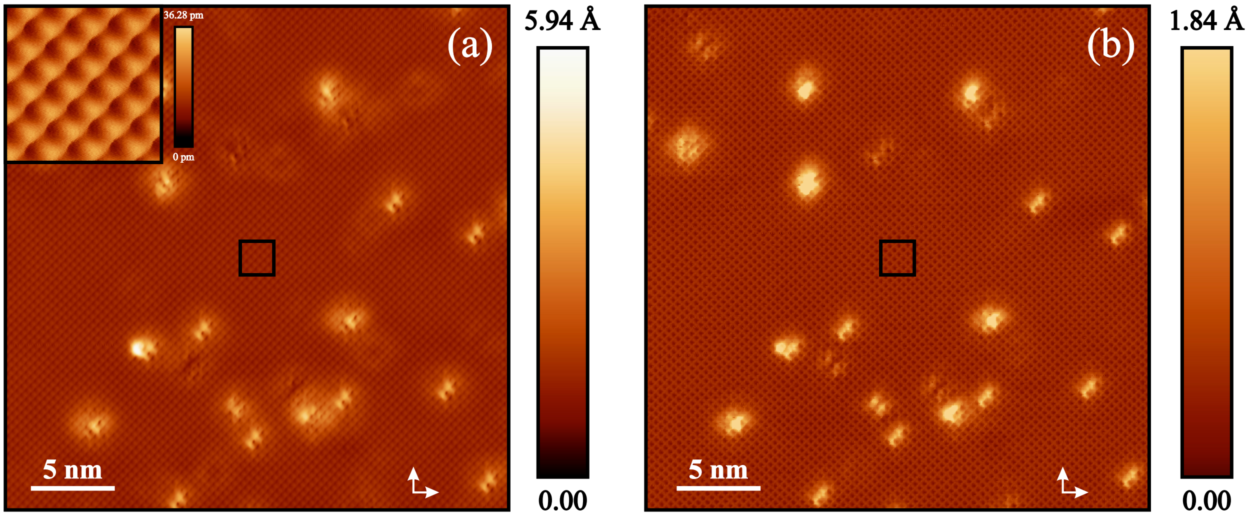

FIG. S2. 30 nm x 30 nm area of atomically resolved constant current mode topography image of LiFeAs ($I_T = 300$ pA) $V_{bias} = +35$ mV (a) and -35 mV (b) measured at $T = 4.8$ K.

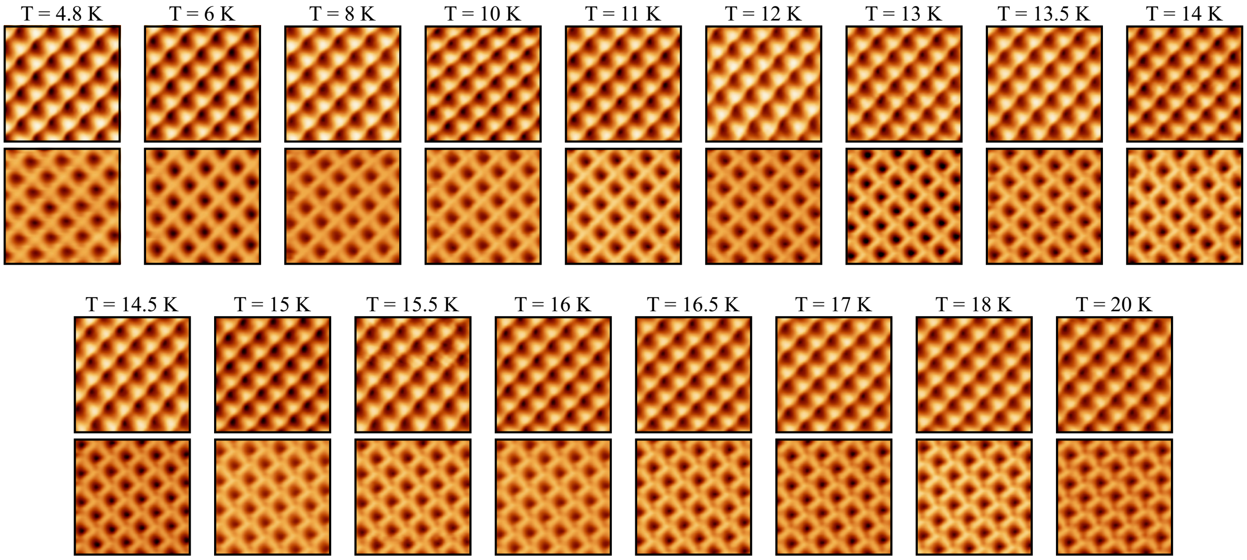

FIG. S3. Topography within the black square of Fig. S2 at all measured temperatures between 4.8 K and 20 K, confirming a constant tip state.

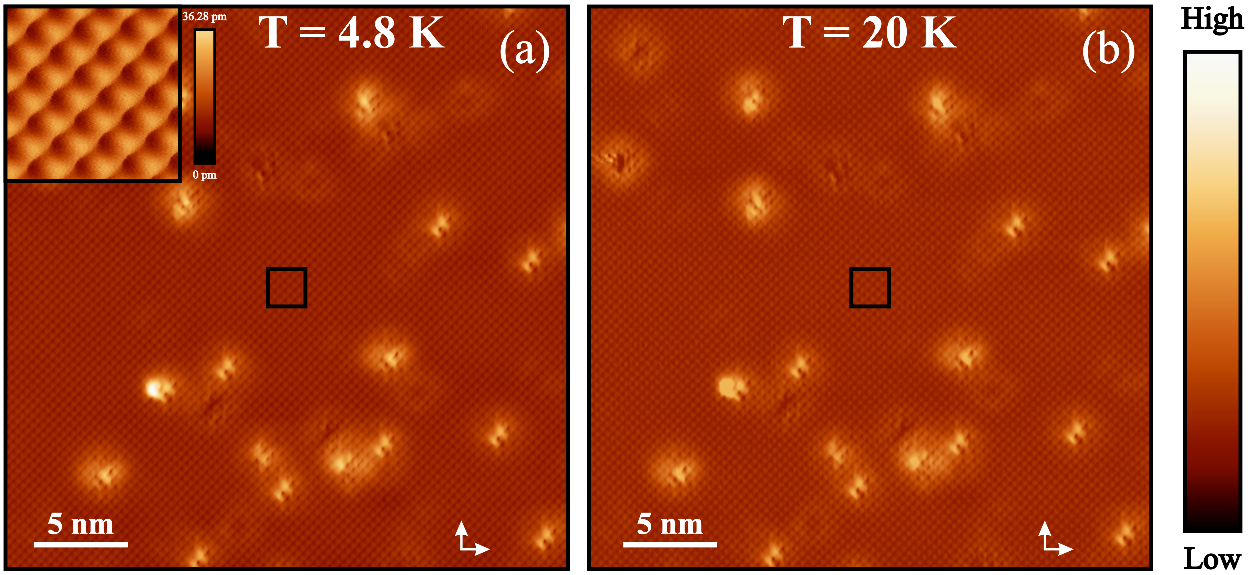

FIG. S4. 30 nm x 30 nm area of atomically resolved constant current mode topography image of LiFeAs (I_T = 300 pA, V_{bias} = +35 mV) measured at T = 4.8 K [(a), same as Fig. S2] and 20 K (b) respectively.
